# High-Resolution ISAR Imaging with Modified Joint Range Spatial-Variant Autofocus and Azimuth Scaling

**DOI:** 10.3390/s20185047

**Published:** 2020-09-05

**Authors:** Jiaqi Wei, Shuai Shao, Hui Ma, Penghui Wang, Lei Zhang, Hongwei Liu

**Affiliations:** 1 National Lab of Radar Signal Processing, Xidian University, Xi’an 710071, China; weijiaqi_0831@126.com (J.W.); shaoshuai_0717@126.com (S.S.); wangpenghui@mail.xidian.edu.cn (P.W.); hwliu@xidian.edu.cn (H.L.); 2 School of Electronics and Communication Engineering, Sun Yat-Sen University, Guangzhou 510275, China; zhanglei57@mail.sysu.edu.cn

**Keywords:** inverse synthetic aperture radar (ISAR), autofocus, azimuth scaling, equivalent rotational center (ERC), minimum entropy optimization lower case

## Abstract

Well-focused and accurately scaled high-resolution inverse synthetic aperture radar (ISAR) images provide a sound basis for feature extraction and target recognition. This paper proposes a novel high-resolution ISAR imaging algorithm, namely modified joint range spatial-variant autofocus and azimuth scaling algorithm (MJAAS). After motion compensation, the shift of the equivalent rotational center (ERC) of the target destroys the linear relationship between the azimuth chirp rates (ACR) of echo signals and the range coordinates of scattering points, thereby leading to the failure of azimuth scaling. Accordingly, a new joint equivalent rotational center position and effective rotational velocity (JERCP-ERV) signal model is established, serving as the basis of MJAAS. By recourse to the Davidon-Fletcher-Powell (DFP) algorithm, MJAAS can jointly estimate the ERCP and ERV by solving a minimum entropy optimization problem, so as to simultaneously achieve accurate azimuth scaling and range spatial-variant autofocus, which further improves the image focusing performance. MJAAS is not restricted by the modes of motion errors (coherent or non-coherent) and the motion compensation methods, so it can be widely applied to real data with the advantages of strong practicality and high accuracy. Extensive experimental results based on both simulated and real data are provided to corroborate the effectiveness of the proposed algorithm.

## 1. Introduction

Inverse synthetic aperture radar (ISAR) can image non-cooperative targets and obtain high-resolution two-dimension (2-D) images, serving as an effective tool for radar target recognition with broad applications in civilian and military fields [1,2,3,4,5,6]. The Range-Doppler (RD) algorithm is usually adopted to generate the target’s ISAR images [7], but the obtained images only reveal the Doppler information of the target in the azimuth dimension rather than the information about the actual size of the target. In order to better identify the target, it is necessary to perform the azimuth scaling of the target. However, motion compensation is a prerequisite for azimuth scaling regarding a set of echo data. Motion compensation algorithms can realize error correction by adjusting the phase information of the echo signal, but most of them inevitably cause the deviation of the target’s rotational center, which impedes the subsequent azimuth scaling. In addition, mainly aimed at the same motion errors of all scattering points on the target, the existing motion compensation algorithms neglect the range spatial-variant phase errors of different scattering points caused by the target’s rotational motion, producing defocused ISAR images. Therefore, it is of vital importance to develop an ISAR imaging algorithm that can jointly achieve accurate azimuth scaling and range spatial-variant autofocus.

The commonly used ISAR azimuth scaling algorithms fall into three main categories. The first is the trajectory tracking method [8] which uses the target’s motion information measured by the narrow band radar to fit the trajectory and then calculates the total rotational angle of the target relative to the radar line of sight (RLOS), so as to achieve azimuth scaling. Due to the large tracking errors made by the narrow band radar, this method offers relatively low accuracy. The second one is the slope-based method [9]. Though intuitionistic and clear, it demands the prior information about the target’s geometric features and has high requirements for the shape of the target on the image used for scaling, thus, its scope of application is narrow. The third is the azimuth chirp rates (ACR) estimation method [10]. It estimates the ACR of echo signals and solves the linear coefficient according to the linear relationship between ACR and range coordinates of the scattering points. The effective rotational velocity (ERV) of the target can be obtained by the estimated linear coefficient, and therefore, the azimuth scaling can be achieved. This kind of method has been widely applied because of its small computation and high accuracy. However, it is easily affected by motion compensation algorithms. Once the motion compensation algorithm destroys the linear relationship between the ACR and the range coordinates of the scattering points, the method fails. Therefore, it is necessary to develop an azimuth scaling algorithm to ensure that the echo signal can still achieve accurate azimuth scaling after completing motion compensation.

The motion compensation can be divided into two parts: range alignment and phase autofocus. Generally, the range alignment is completed first followed by the phase autofocus. The commonly used range alignment algorithms include the maximum cross-correlation method [11] and the minimum entropy method [12]. This paper employs an improved maximum cross-correlation method (IMCM) [8] to achieve range alignment. This algorithm works out the weighted sum of all the aligned envelopes, which replaces the adjacent echoes in the traditional maximum cross-correlation method as the reference in the cross-correlation operation; thus, it has good robustness. In terms of phase autofocus, methods such as the multiple dominant scatterers method [13,14], the phase gradient autofocus algorithm (PGA) [15,16,17] and the minimum entropy method [18,19] are frequently adopted. In this paper, we use PGA to realize phase autofocus in light of its strong practicality and low computational complexity. The algorithm compensates the phase errors of the whole echo signal by calculating the phase gradient of the dominant scatterer. That is to say, the dominant scatterer (usually the composite point of multiple dominant scatterers) is regarded as the rotational center and recorded as the equivalent rotational center (ERC). It is easy to know that, after the processing of PGA, the ACR of the echo signal is no longer linear with the range coordinates of the scattering points, which renders the azimuth scaling algorithm based on the ACR invalid. In addition to the cascaded motion compensation method above, a joint translational motion compensation algorithm is proposed in [20]. The algorithm is highly robust against noise but not suitable for the non-coherent mode of the motion errors; thus, its practicality is poor. Furthermore, the aforementioned motion compensation algorithms only consider the same motion errors of all scattering points and neglect the exclusive range spatial-variant phase errors of different scattering points caused by the target’s rotational motion [21,22]. Thus, it is desired to advance an algorithm by jointly estimating the equivalent rotational center position (ERCP) and effective rotational velocity (ERV), which can achieve the range spatial-variant autofocus while precisely accomplishing azimuth scaling.

Inspired by the aforementioned challenges, this paper presents a novel high-resolution ISAR imaging algorithm, namely the modified joint range spatial-variant autofocus and azimuth scaling algorithm (MJAAS). The main contributions of the proposed algorithm are as follows:

(1) By analyzing the processing results of the motion compensation algorithm, a novel joint equivalent rotational center position and effective rotational velocity (JERCP-ERV) signal model is established. It takes into account the deviation of the ERC caused by the motion compensation algorithm, and jointly estimates the ERCP and ERV, so as to achieve the modification of the ERCP.

(2) Based on the JERCP-ERV signal model, MJAAS is proposed. In this technique, the Davidon-Fletcher-Powell (DFP) [23] algorithm is used to solve a minimum entropy optimization problem, thereby jointly realizing the accurate estimation of ERCP and ERV. Furthermore, the precise range spatial-variant autofocus and azimuth scaling can be simultaneously completed on the basis of the estimation results. It is noteworthy that the application of MJAAS is not limited to certain types of motion compensation algorithms. MJAAS can jointly achieve accurate azimuth scaling and range spatial-variant autofocus by estimating the corresponding ERCP and ERV with regard to different motion compensation algorithms.

Based on MJAAS as well as combined with IMCM and PGA, a complete high-resolution ISAR imaging framework is formed, which is not restrained by the modes of motion errors (coherent or non-coherent) of the target. With low computational complexity, the framework has the advantages of strong practicality and high efficiency.

This paper is organized as follows. Section 2 introduces the echo signal model, motion compensation algorithms and azimuth scaling algorithms, and analyzes the influence of the motion compensation algorithm on the azimuth scaling algorithm. On this basis, a novel JERCP-ERV signal model is established. Section 3 proposes the MJAAS algorithm to jointly achieve accurate range spatial-variant autofocus and azimuth scaling. Section 4 provides the experimental results of both simulated and real data to verify the effectiveness of the proposed algorithm. The paper ends with a brief conclusion in Section 5.

## 2. Signal Model and Related Work

### 2.1. Signal Model

**Figure 1:**
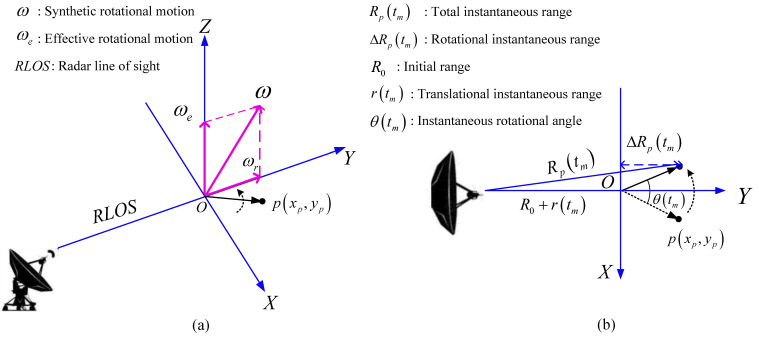
Inverse synthetic aperture radar (ISAR) model© 2020 IEEE. (**a**) Three-dimensional (3-D) scenario geometry model. (**b**) Simplified two-dimensional (2-D) scenario geometry model.

The three-dimension (3-D) ISAR imaging geometry model is shown in Figure 1a, where the radar line of sight (RLOS) direction from the radar to the rotational center of the target O is defined as the Y-axis. Point p is a scattering point on the target whose coordinate on the XOY plane is $\left(x_{p},y_{p}\right)$. The synthetic rotational motion ω of the target can be divided into two orthogonal parts: one along the Y-axis, recorded as $\omega_{r}$, and the other along the Z-axis, recorded as effective rotational motion $\omega_{e}$. According to the Y-axis and Z-axis, the X-axis is determined using the right-hand rule so that the Cartesian coordinate system $\left(X,Y,Z\right)$ can be established as shown in Figure 1a. Given that $\omega_{r}$ does not change the slant distance between the radar and the target, it makes no contribution to ISAR imaging. $\omega_{e}$ is the only effective part, serving as the essential source of ISAR imaging. In this paper, it is assumed that the direction and magnitude of $\omega_{e}$ are constant, that is, the target makes 2-D uniform rotational motion. Therefore, the 3-D imaging geometry model in Figure 1a can be simplified to the 2-D imaging model shown in Figure 1b, where $R_{p}\left(t_{m}\right)$ represents the instantaneous range between radar and p; $\Delta R_{p}\left(t_{m}\right)$ is the instantaneous range between the scattering point p and the radar caused by rotational motion; $R_{0}$ represents the initial range between the rotational center of the target O and the radar; $r\left(t_{m}\right)$ is the instantaneous range between the target and the radar caused by translational motion, which remains the same for all scattering points; and $\theta \left(t_{m}\right)$ is the instantaneous rotational angle of the target along Z-axis at $t_{m}$. The radar is assumed to transmit a linear frequency modulated (LFM) signal. After preprocessing (range compression and de-modulation to the baseband), the received signal of the pth scattering point can be given by Equation (1).

(1) $$s_{p}\left(\hat{t};t_{m}\right)=\sigma_{p}\cdot \mathrm{sinc}\left[B\left(\hat{t}-\frac{2R_{p}\left(t_{m}\right)}{c}\right)\right]\cdot \exp\left[-j\frac{4\pi f_{c}\cdot R_{p}\left(t_{m}\right)}{c}\right]$$

where $\hat{t}$ and $t_{m}$ represent fast time and slow time; $\sigma_{p}$ denotes the scattering coefficient of the scattering point p; B and $f_{c}$ refer to the bandwidth and carrier frequency of the transmitted signal; c is the light velocity; and the instantaneous range between radar and p is represented by $R_{p}\left(t_{m}\right)$, which is expressed as Equation (2) [10]:

(2) $$\begin{array}{ll} R_{p}\left(t_{m}\right) & =R_{0}+r\left(t_{m}\right)+\Delta R_{p}\left(t_{m}\right)\\ & \approx R_{0}+r\left(t_{m}\right)+x_{p}\cdot \sin\left[\theta \left(t_{m}\right)\right]+y_{p}\cdot \cos\left[\theta \left(t_{m}\right)\right] \end{array}$$

where $x_{p}$ and $y_{p}$ are the abscissa and ordinate of p on the XOY plane, respectively. $\Delta R_{p}\left(t_{m}\right)$ is expanded using the Tayler series, which can be approximated as Equation (3):

(3) $$\begin{array}{ll} \Delta R_{p}\left(t_{m}\right) & \approx x_{p}\cdot \theta \left(t_{m}\right)+y_{p}\cdot \left[1-\theta^{2}\left(t_{m}\right)/2\right]\\ & =x_{p}\cdot \omega_{e}\cdot t_{m}+y_{p}\cdot \left(1-\omega_{e}^{2}\cdot t_{m}^{2}/2\right) \end{array}$$

where $\cos\left[\theta \left(t_{m}\right)\right]\approx 1-\theta^{2}\left(t_{m}\right)/2$ and $\sin\left[\theta \left(t_{m}\right)\right]\approx \theta \left(t_{m}\right)=\omega_{e}\cdot t_{m}$. Assigning Equations (2) and (3) to (1), Equation (1) can be rephrased as Equation (4):

(4) $$\begin{array}{ll} s_{p}\left(\hat{t};t_{m}\right) & =\sigma_{p}\cdot \mathrm{sinc}\left\{B\left\{\hat{t}-\frac{2\left[R_{0}+r\left(t_{m}\right)+\Delta R_{p}\left(t_{m}\right)\right]}{c}\right\}\right\}\\ & \cdot \exp\left\{-j\frac{4\pi f_{c}\cdot \left[R_{0}+y_{p}+r\left(t_{m}\right)+x_{p}\omega_{e}t_{m}-y_{p}\omega_{e}^{2}t_{m}^{2}/2\right]}{c}\right\} \end{array}$$

where $\mathrm{sinc}\left\{\cdot \right\}$ represents the range envelope term and $\exp\left\{\cdot \right\}$ the phase term. In the envelope term, $r\left(t_{m}\right)$ causes the range shift. The migration through resolution cells (MTRC) caused by $\Delta R_{p}\left(t_{m}\right)$ can be neglected since it is usually less than half of a range bin in a short coherent processing interval (CPI). Even if MTRC exists, there are some algorithms available to eliminate it [24]. For the phase term, $r\left(t_{m}\right)$, which is identical for all scattering points, leads to phase errors. Due to the coupling of the quadratic term of $t_{m}$ and the ordinate $y_{p}$ of the scattering point p, the range spatial-variant phase errors arise. In order to create high-resolution ISAR images, apart from correcting the translational motion errors caused by the range spatial-variant phase errors caused by the quadratic term of $t_{m}$ also need to be accurately compensated. Note that, since the quadratic term of $t_{m}$ contains the target’s effective rotational velocity (ERV) $\omega_{e}$, it can be used to estimate $\omega_{e}$ so as to achieve the azimuth scaling of ISAR images [13]. Based on the above analysis, with MTRC and the constant phase term neglected, the discrete form of Equation (4) can be expressed as Equation (5):

(5) $$\begin{array}{ll} s_{p}\left(n;m\right) & =\sigma_{p}\cdot \mathrm{sinc}\left\{B\left\{n\cdot \Delta \hat{t}-\frac{2\left[R_{0}+y_{p}+r\left(m\cdot \Delta t_{m}\right)\right]}{c}\right\}\right\}\\ & \cdot \exp\left\{-j\frac{4\pi f_{c}\cdot \left[r\left(m\cdot \Delta t_{m}\right)+x_{p}\omega_{e}\cdot m\cdot \Delta t_{m}-y_{p}\omega_{e}^{2}{\left(m\cdot \Delta t_{m}\right)}^{2}/2\right]}{c}\right\} \end{array}$$

where n and m refer to the indexes of the range bin and azimuth bin, respectively, −N/2≤n≤N/2−1, −M/2≤m≤M/2−1; N and M denote the total numbers of the range bin and azimuth bin, respectively; and $\Delta \hat{t}$ and $\Delta t_{m}$ represent the sampling interval of fast time and slow time, respectively.

### 2.2. Related Work

It can be seen from Equation (5) that the motion errors of the echo signal lie in the envelope term and phase term. Therefore, the motion compensation consists of two parts: range alignment and phase adjustment. In addition, instead of the information about the actual size of the target, the ISAR image obtained by the range-Doppler (RD) algorithm displays the Doppler information of each scattering point in the azimuth dimension, which has an adverse effect on the subsequent feature extraction and target recognition. Therefore, for a complete ISAR imaging framework, in addition to motion compensation, it is necessary to carry out accurate azimuth scaling of the RD image. This section will briefly introduce the commonly used motion compensation and azimuth scaling algorithms in practice.

#### 2.2.1. Range Alignment

Range alignment, the premise of phase autofocus, is to remove the $r\left(m\cdot \Delta t_{m}\right)$ in the envelope term of Equation (5). Various algorithms such as the maximum cross-correlation method and the minimum entropy method have been proposed to achieve range alignment, while this paper employs an improved maximum cross-correlation method. A brief introduction to the principle of this algorithm is given in what follows.

The traditional maximum cross-correlation method calculates the cross-correlation coefficient between the envelopes of two adjacent echoes and determines the delay value through peak search so as to achieve the alignment of adjacent pulses. However, since envelope drifting and jump errors often occur when processing the real data [8], the robustness of the algorithm is poor. To solve this problem, an improved maximum cross-correlation method (IMCM) is proposed. When aligning a certain envelope, this algorithm uses the weighted sum of all the aligned envelopes rather than the previous one envelope as the reference for cross-correlation processing. The employment of multiple echoes helps avoid the envelope drifting and jump errors, thereby improving the robustness of the range alignment.

#### 2.2.2. Phase Adjustment

A phase adjustment is performed to eliminate the motion error in the phase term of Equation (5). A large number of phase adjustment algorithms have been proposed, including the multiple dominant scatterers synthesis method, PGA and the minimum entropy method. In the experiments of this paper, we use PGA to realize the phase adjustment. It is worth noting that the above algorithms only consider the same phase errors of each scattering point but neglect the exclusive spatial-variant phase errors. That is to say, these algorithms can only eliminate $r\left(m\cdot \Delta t_{m}\right)$ in the phase term of Equation (5), but find it difficult to eliminate $y_{p}\omega_{e}^{2}{\left(m\cdot \Delta t_{m}\right)}^{2}/2$. In addition, they will cause deviation of equivalent rotational center (ERC), which distorts the subsequent azimuth scaling. Next, we will give a brief introduction to PGA to show its influence on the azimuth scaling algorithm. PGA is an iterative algorithm with operations in every iteration repeated, thus, what follows only introduces the process of one iteration to illustrate its rationale.

In general, PGA firstly extracts 20–30 dominant scatterers to calculate the phase gradient. For clarity, the principle of the algorithm is elucidated by one extracted dominant scatterer, and the same applies to the case of multiple dominant scatterers. For instance, the scattering point p in Equation (5) is used as the dominant scatterer. Mark that the phase of the scattering point p is $\phi_{p}\left(m\right)$ and $m\cdot \Delta t_{m}$ is m. Then, $\phi_{p}\left(m\right)$ can be written as Equation (6):

(6) $$\phi_{p}\left(m\right)=\frac{4\pi f_{c}}{c}\cdot \left[r\left(m\right)+x_{p}\omega_{e}m-y_{p}\omega_{e}^{2}m^{2}/2\right].$$

the cyclic shift of the complex image corresponding to the dominant scatterer p is carried out to move the peak value of the dominant scatterer p to the center of the image (i.e., zero Doppler). Then, $\phi_{p}\left(m\right)$ can be rewritten as Equation (7):

(7) $$\phi_{p}\left(m\right)=\frac{4\pi f_{c}}{c}\cdot \left[r\left(m\right)-\xi_{p}\left(m\right)\right]$$

where $\xi_{p}\left(m\right)=y_{p}\omega_{e}^{2}m^{2}/2$. Then, the phase difference as shown in Equation (8) can be obtained by the conjugate multiplication of the mth and the m−1th echoes:

(8) $$\Delta \phi_{p}\left(m\right)=\phi_{p}\left(m\right)-\phi_{p}\left(m-1\right)=\frac{4\pi f_{c}}{c}\cdot \left[\Delta r\left(m\right)+\Delta \xi_{p}\left(m\right)\right]$$

where $\Delta r\left(m\right)=r\left(m\right)-r\left(m-1\right)$ and $\Delta \xi_{p}\left(m\right)=\xi_{p}\left(m\right)-\xi_{p}\left(m-1\right)$. Let $\Delta \phi_{p}\left(-M/2\right)=0$ and m=−M/2,⋯,M/2−1. Next, Equation (9) is used to calculate the phase gradient $\varphi_{p}\left(i\right)$ ($\varphi_{p}\left(i\right)$ is the phase error of the ith echo signal needed to be corrected for each range bin):

(9) $$\varphi_{p}\left(i\right)=\sum_{m=-M/2}^{i}\Delta \phi_{p}\left(m\right).$$

Finally, the phase error is rectified by compensating the whole ISAR echo signal with the help of $\varphi_{p}\left(i\right)$ (i=−M/2,⋯,M/2−1). The above is the basic principle of PGA. However, according to Equation (8), in addition to the translational motion error $\Delta r\left(m\right)$, the range spatial-variant phase error $\Delta \xi_{p}\left(m\right)$ also exists in $\Delta \phi_{p}\left(m\right)$. When using $\varphi_{p}\left(i\right)$ for phase compensation, $\Delta r\left(m\right)$ can be precisely compensated. Nevertheless, since $\Delta \xi_{p}\left(m\right)$ is spatial-variant, its presence in the echo signal will render the image defocused. It is assumed that the distance range of the target support area is $\left[-\bar{M}/2,\bar{M}/2-1\right]$ and the rotational center of the target is located at the origin $\bar{M}<M$. After compensating the whole echo signal by $\varphi_{p}\left(i\right)$, the distance range of the target support area in the phase term becomes $\left[-\bar{M}/2-y_{p},\bar{M}/2-y_{p}-1\right]$ since $\Delta \xi_{p}\left(m\right)$ contains the ordinate $y_{p}$ of the scattering point p. At this time, the scattering point p is equivalent to the rotational center of the target (the ordinate is 0), thus, it is marked as ERC. The above analysis suggests that the position of the rotational center shifts after compensation, thus, the relationship between the azimuth chirp rates (ACR) of the echo signal and the range coordinates of the scattering points $\left[-\bar{M}/2,\bar{M}/2-1\right]$ is no longer a linear one, which severely distorts the subsequent azimuth scaling. This explains why it is of vital importance to propose an algorithm which can simultaneously compensate the range spatial-variant phase error and overcome the distortion of azimuth scaling caused by the equivalent rotational center position (ERCP) deviation. It should be noted that though the above analysis is based on PGA, other phase autofocus algorithms have the same problem.

#### 2.2.3. Azimuth Scaling

Among the existing azimuth scaling algorithms, the one based on ACR has been frequently adopted thanks to its high accuracy. It can be seen from Equation (6) that the ACR $\alpha_{n}$ of the nth range bin is Equation (10) delete extra space:

(10) $$\alpha_{n}=\frac{2\omega_{e}^{2}}{\lambda }\cdot y_{n}=K\cdot y_{n}$$

where $\lambda =c/f_{c}$ denotes radar wavelength and $y_{n}$ is the coordinate of the nth range bin. It can be seen from Equation (10) that there is a simple linear relationship between $\alpha_{n}$ and $y_{n}$. $y_{n}$ can be obtained by multiplying the range resolution by the range bin number n. As long as the ACR is estimated, the linear coefficient K can be obtained. Then, according to $\omega_{e}={\left(\lambda \left|K\right|/2\right)}^{1/2}$, the ERV can be obtained and the azimuth scaling can be realized. The above is the basic principle of the azimuth scaling algorithm based on ACR. However, from the analysis of the autofocus algorithm in the previous section, the ERC in the phase term shifts after phase compensation. Therefore, the solution of multiplying range resolution by the range bin number n to obtain $y_{n}$ in $\alpha_{n}$ no longer works. If the position of the ERC is not corrected, the wrongly estimated $\omega_{e}$ will result in the distortion of azimuth scaling. Nevertheless, few existing azimuth scaling algorithms consider this problem.

In the next subsection, we establish a more refined signal model, called joint equivalent rotational center position and effective rotational velocity signal model (JERCP-ERV). It includes two parameters, namely the equivalent rotational center position (ERCP) and the effective rotational velocity (ERV), making it possible to eliminate the estimation error of ERV caused by the shift of ERCP.

### 2.3. JERCP-ERV Signal Model

Equation (5) is the echo signal of the scattering point p. After motion compensation described in the previous section, the compensation result ${\tilde{s}}_{p}\left(n;m\right)$ can be expressed as Equation (11):

(11) $$\begin{array}{ll} {\tilde{s}}_{p}\left(n;m\right) & =\sigma_{p}\cdot \mathrm{sinc}\left\{B\left\{n\cdot \Delta \hat{t}-\frac{2\left[R_{0}+y_{p}\right]}{c}\right\}\right\}\\ & \cdot \exp\left\{-j\frac{4\pi f_{c}\cdot \left[x_{p}\omega_{e}\cdot m\cdot \Delta t_{m}-\left(l_{n,p}-\beta \right)\cdot \Omega \cdot {\left(m\cdot \Delta t_{m}\right)}^{2}\right]}{c}\right\} \end{array}$$

where β denotes the range coordinate of the ERC in the range scene; $\Omega =\omega_{e}^{2}/2$, $l_{n,p}=n_{p}\cdot c/2B$ and $n_{p}$ refers the range bin index of the scattering point p. Equation (11) is the novel JERCP-ERV signal model with two unknown parameters proposed in this paper, the equivalent rotational center position (ERCP) and the effective rotational velocity (ERV), the joint estimation of which can be realized according to the signal model. Assuming that there are a total of P scattering points on the target, the total echo signal of the target after motion compensation can be expressed as Equation (12):

(12) $$\tilde{S}\left(n;m\right)=\sum_{p=0}^{P-1}{\tilde{s}}_{p}\left(n;m\right).$$

## 3. The Proposed Methodology

In this section, a novel modified joint range spatial-variant autofocus and azimuth scaling algorithm (MJAAS) is proposed based on the analysis above. It can realize range spatial-variant autofocus while achieving accurate azimuth scaling so as to further improve the focusing performance of the image.

### 3.1. The Establishment of Objective Function

For clarity, we define a parameter vector, $\Psi =\left[\beta ,\Omega \right]$. After compensation with $\hat{\Psi }=\left[\hat{\beta },\hat{\Omega }\right]$, the complex ISAR image $g\left(n;k;\hat{\Psi }\right)$ as shown in Equation (13) can be obtained by processing Equation (12) with azimuth fast Fourier transform (FFT):

(13) $$\begin{array}{ll} g\left(n;k;\hat{\Psi }\right) & =\frac{1}{M}\sum_{m=-M/2}^{M/2-1}\left\{\exp\left(-j\frac{2\pi }{M}mk\right)\cdot \tilde{S}\left(n;m\right)\cdot \exp\left\{-j\frac{4\pi }{\lambda }\cdot \left[\left(l_{n}-\hat{\beta }\right)\cdot \hat{\Omega }\cdot {\left(m\cdot \Delta t_{m}\right)}^{2}\right]\right\}\right\}\\ & =\frac{1}{M}\sum_{m=-M/2}^{M/2-1}\left\{\exp\left(-j\frac{2\pi }{M}mk\right)\cdot \hat{S}\left(n;m\right)\right\} \end{array}$$

where $\hat{\Psi }=\left[\hat{\beta },\hat{\Omega }\right]$ is the estimated value of $\Psi =\left[\beta ,\Omega \right]$; The result of compensating $\tilde{S}\left(n;m\right)$ with $\hat{\Psi }$ is $\hat{S}\left(n;m\right)$; $l_{n}=n\cdot c/2B$, n is the range bin index, and −N/2≤n≤N/2−1; k is the Doppler bin index; and −M/2≤k≤M/2−1. Equation (13) indicates that the accurate estimation of $\hat{\Psi }=\left[\hat{\beta },\hat{\Omega }\right]$ can ensure a well-focused and accurately scaled ISAR image. Generally speaking, parameter estimation can be realized by solving an unconstrained optimization problem. It is widely acknowledged that image entropy (IE) is an effective index of evaluating image quality. IE is therefore chosen as the objective function to solve the unknown parameters $\hat{\Psi }=\left[\hat{\beta },\hat{\Omega }\right]$ in this paper. According to the definition of IE, it can be expressed as Equation (14):

(14) $$IE=\ln E_{g}-\frac{1}{E_{g}}\sum_{n=-N/2}^{N/2-1}\sum_{k=-M/2}^{M/2-1}\left(\mu \cdot \ln\mu \right)$$

where $\mu ={\left|g\left(n;k;\hat{\Psi }\right)\right|}^{2}$ and $E_{g}=\sum_{n=-N/2}^{N/2-1}\sum_{k=-M/2}^{M/2-1}\mu$ is the image intensity.

From Equation (14) we can see that since the phase error compensation term $\exp\left\{-j\frac{4\pi }{\lambda }\cdot \left[\left(l_{n}-\hat{\beta }\right)\cdot \hat{\Omega }\cdot {\left(m\cdot \Delta t_{m}\right)}^{2}\right]\right\}$ is performed on the phase of $\tilde{S}\left(n;m\right)$, the signal energy holds constant according to the Parseval theorem [25]. Therefore, $E_{g}$ is independent of $\hat{\Psi }$. The estimate of $\hat{\Psi }=\left[\hat{\beta },\hat{\Omega }\right]$ is obtained by minimizing the image entropy, expressed as Equation (15):

(15) $$\langle \hat{\beta },\hat{\Omega }\rangle =\arg\underset{\hat{\beta },\hat{\Omega }}{\min}\left\{IE\right\}.$$

### 3.2. Optimal Parameters Estimation

Many algorithms are now available to solve Equation (15), such as the gradient descent algorithm (GDA) [26] and quasi-Newton method [27]. As a highly efficient quasi-Newton algorithm, the Davidon-Fletcher-Powell (DFP) algorithm [23] is selected to solve Equation (15) in this paper. It requires to calculate the gradient of the objective function corresponding to the unknown parameters, thus, the gradient of IE with regard to $\hat{\Psi }=\left[\hat{\beta },\hat{\Omega }\right]$ can be derived as Equation (16):

(16) $$\nabla IE\left[\hat{\Psi }\right]=\left[\frac{\partial IE}{\partial \hat{\beta }},\frac{\partial IE}{\partial \hat{\Omega }}\right]$$

the detailed derivation process of $\nabla IE\left[\hat{\Psi }\right]$ is given in Appendix A. In addition to gradient, an approximate matrix H is used to replace the inverse of Hessian matrix of the objective function in DFP. The updated formula of H is given in Appendix B. With $\nabla IE\left[\hat{\Psi }\right]$ and H acquired, the optimal values of the unknown parameters $\hat{\Psi }=\left[\hat{\beta },\hat{\Omega }\right]$ can be obtained by iterative processing along the search direction $\left\{-H\cdot \nabla IE\left[\hat{\Psi }\right]\right\}$. In addition, $\nabla IE\left[\hat{\Psi }\right]$ and H are updated in each iteration according to Equations (16) and (23). After obtaining $\nabla IE\left[{\hat{\Psi }}^{\left[u\right]}\right]$ and $H^{\left[u\right]}$, the updated formula of ${\hat{\Psi }}^{\left[u\right]}$ can be expressed as Equation (17):

(17) $${\hat{\Psi }}^{\left[u+1\right]}={\hat{\Psi }}^{\left[u\right]}-\eta^{\left[u\right]}\cdot H^{\left[u\right]}\cdot \nabla IE\left[{\hat{\Psi }}^{\left[u\right]}\right]$$

where $\eta^{\left[u\right]}=\left[\eta_{\hat{\beta }}^{\left[u\right]},\eta_{\hat{\Omega }}^{\left[u\right]}\right]$ stands for the search step vector which can be accurately estimated by one-dimension (1-D) search algorithms such as the Armijo criterion [28] and golden section method [29], so as to prevent the objective function from falling into the local optimal solution. Without prior information, the initial value of the unknown parameter is usually set as ${\hat{\Psi }}^{\left[0\right]}=\left[0,0\right]$. In general, IE is able to converge when U is less than 10. It is worth noting that in order to ensure the robustness and astringency of the proposed algorithm, we apply the idea of coordinate descent when estimating $\hat{\Psi }=\left[\hat{\beta },\hat{\Omega }\right]$, that is, fixing one parameter and estimating another one [20]. Through the above processing, the estimated optimal parameter ${\hat{\Psi }}_{opt}=\left[{\hat{\beta }}_{opt},{\hat{\Omega }}_{opt}\right]$ can be obtained. In this way, we can create a well-focused and accurately-scaled ISAR image according to Equation (13).

According to the analysis above, for the proposed algorithm, only the gradient of the objective function needs to be solved, thus, the computational complexity of the proposed algorithm mainly lies in the calculation of $\nabla IE\left[{\hat{\Psi }}^{\left[u\right]}\right]$ and the search of $\eta^{\left[u\right]}$. Equation (16) indicates that the computational complexity of gradient calculation mainly depends on the azimuth FFT. The computational complexity is represented by the number of complex multiplication, and that of FFT corresponding to M points is $O\left(M\cdot \log_{2}M\right)$. It is assumed that the iterative number of searching $\eta^{\left[u\right]}$ is V, then the computational complexity of the proposed algorithm in one iteration is $O\left(2\cdot \left(V+1\right)\cdot N\cdot \left(M\cdot \log_{2}M\right)\right)$, thus, its total computational complexity is $O\left(U\cdot \left(2\cdot \left(V+1\right)\cdot N\cdot \left(M\cdot \log_{2}M\right)\right)\right)$.

At present, the commonly used ISAR azimuth scaling algorithms fall into three main categories: the trajectory tracking method (TTM) in reference [8], the slope-based method (SBM) in reference [9] and the azimuth chirp rates estimation method (ACREM) in reference [10]. TTM uses the target’s motion information measured by the narrow band radar to fit the trajectory, and then calculates the total rotational angle of the target relative to the radar line of sight (RLOS), so as to achieve azimuth scaling. Due to the large tracking errors caused by the narrow band radar, this method offers relatively low accuracy. Completely based on the target image, the SBM method only needs to estimate the slope of the two characteristic lines of the target to complete the azimuth scaling. However, it has a limited application since it demands the prior information about the target’s geometric features and has high requirements for the shape of the target on the image used for scaling. The ACREM method uses the azimuth chirp rates (ACR) information contained in the signal to estimate the effective rotational velocity (ERV) of the target directly, thus realizing the azimuth scaling. This kind of method has been widely applied because of its small computation and high accuracy. However, it is easily affected by motion compensation algorithms. Once the motion compensation algorithm destroys the linear relationship between the ACR and the range coordinates of the scattering points, the method will fail. Next, we compare the pros/cons of the proposed algorithm and the above three algorithms through Table 1.

**Figure 2:**
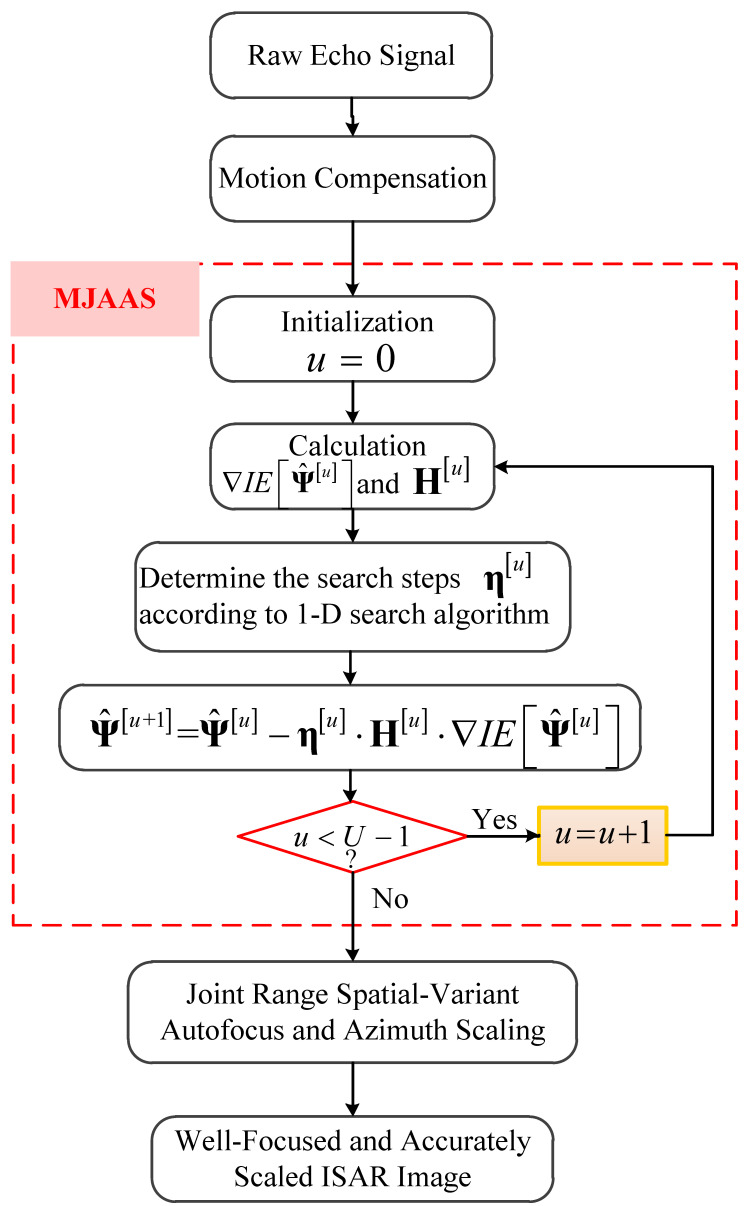
Flowchart of the proposed algorithm.

From Table 1, we can see that each algorithm has its own pros/cons, but all things considered, the proposed algorithm is a better choice. A flowchart is given in Figure 2 to elucidate the proposed algorithm.

## 4. Experiments and Analyses

In this section, based on the proposed MJAAS algorithm as well as combined with improved maximum cross-correlation method (IMCM) and phase gradient autofocus algorithm (PGA), a complete high-resolution ISAR imaging framework, IMCM + PGA + MJAAS (IMCM-PGA-MJAAS), is developed. IMCM+PGA (IMCM-PGA)MJAAS uses IMCM for range alignment and PGA for phase autofocus without azimuth scaling. IMCM + PGA + TAS (IMCM-PGA-TAS) adopts the same motion compensation method as IMCM-PGA, but involves the traditional azimuth scaling algorithm (TAS) based on the ACR to realize azimuth scaling, and the ERV estimated by TAS is used for range spatial-variant autofocus. IMCM-PGA-MJAAS is compared with IMCM-PGA and IMCM-PGA-TAS based on simulated and real data. IMCM-PGA-MJAAS also utilizes the same motion compensation algorithm as the two comparison algorithms. What makes it different is the employment of MJAAS, which can jointly realize accurate range spatial-variant autofocus and azimuth scaling. In addition, we set two modes of motion errors, coherent mode (CM) and non-coherent mode (NCM), and different SNRs to illustrate the superiority of the proposed algorithm in the following experiments.

### 4.1. Simulated Data Experiments

**Figure 3:**
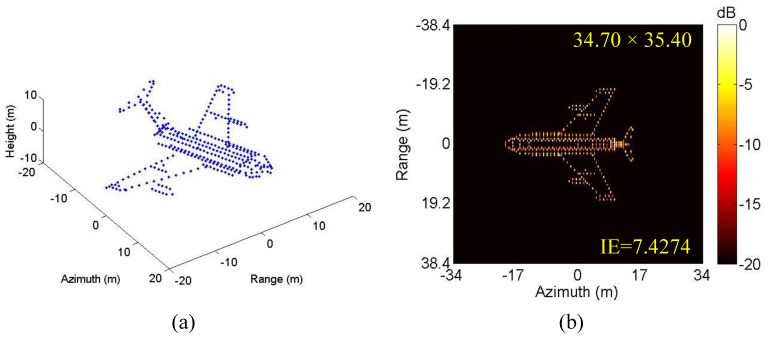
Simulated data of Yak-42 airplane. (**a**) 3-D scattering point model. (**b**) Standard ISAR image.

**Figure 4:**
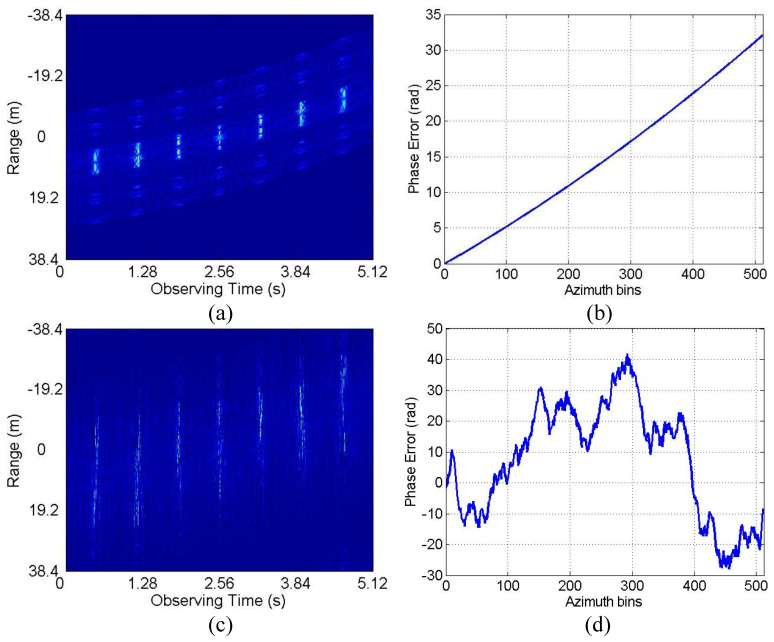
HRRPs and phase errors. CM: (**a**) HRRPs. (**b**) Phase errors. NCM: (**c**) HRRPs. (**d**) Phase errors.

In this case, the 3-D scattering point model of Yak-42 airplane as shown in Figure 3a, which is 34.68 m in length and 35.48 m in width, is used to generate simulated data, and the resulted standard 2-D RD image is shown in Figure 3b. The radar system adopts the de-chirp processing mode with the main simulated parameters shown in Table 2. As mentioned above, echo data high-resolution range profiles (HRRP) generated in CM and NCM are employed to verify the performance of the proposed algorithm, with corresponding envelope waveforms and phase errors shown in Figure 4. In practice, both CM and NCM may occur in the target echo signal received by radar, with the former mainly for the stable motion of the target and the later mainly for the serious vibration of the target. Concerning the actual situation of the echo signal, the real data will be analyzed in detail in the next subsection.

**Figure 5:**
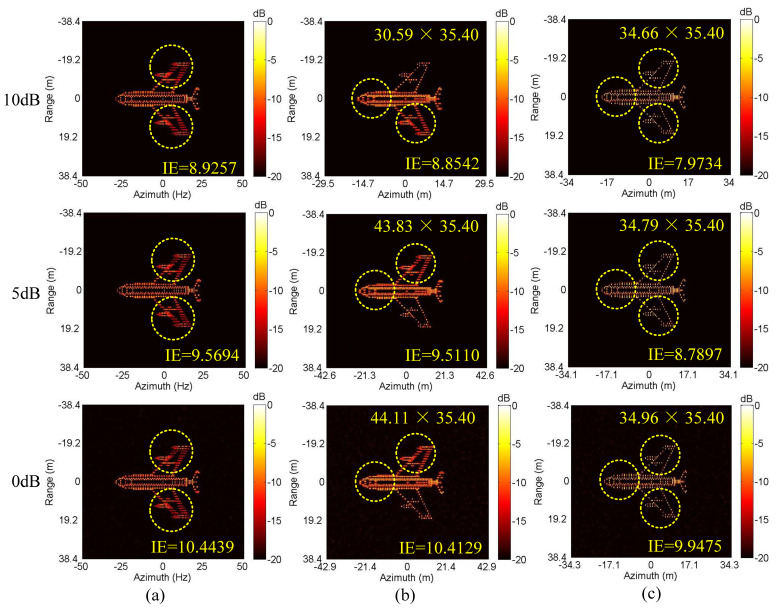
The imaging results of different algorithms at different SNRs corresponding to CM. (**a**) IMCM-PGA. (**b**) IMCM-PGA-TAS. (**c**) IMCM-PGA-MJAAS.

**Figure 6:**
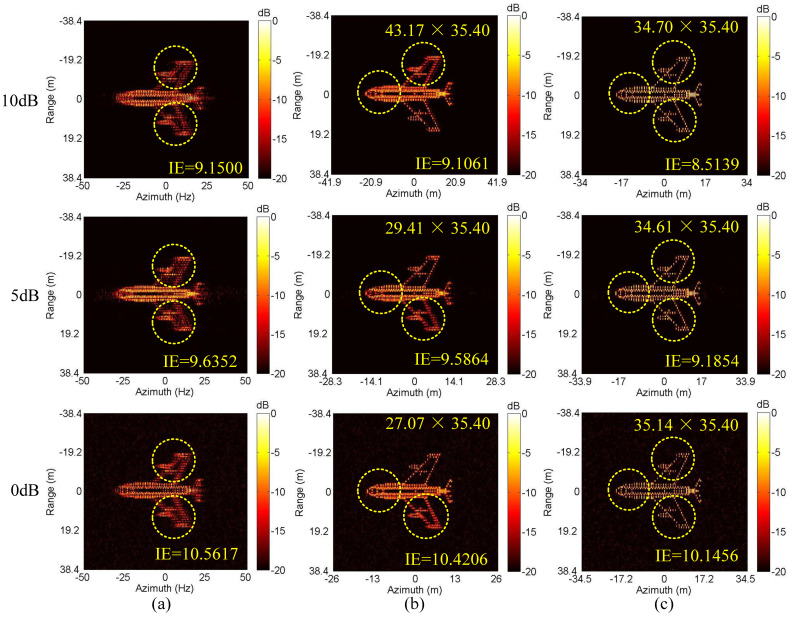
The imaging results of different algorithms at different SNRs corresponding to NCM. (**a**) IMCM-PGA. (**b**) IMCM-PGA-TAS. (**c**) IMCM-PGA-MJAAS.

Next, we conduct the ISAR imaging in CM as shown in Figure 4a,b. Complex Gaussian white noise is added to the data in Figure 4a,b to generate three SNRs (10 dB, 5 dB and 0 dB). Then, ISAR imaging was performed by IMCM-PGA, IMCM-PGA-TAS and IMCM-PGA-MJAAS at different SNRs with corresponding imaging results shown in Figure 5. The imaging results of IMCM-PGA are defocused because it fails to take into account the range spatial-variant phase error caused by the rotational motion of the target. With regards to the imaging results of IMCM-PGA-TAS and the target’s size marked in the figure, there are severe errors in azimuth scaling compared with the real size of the target. The distortion of azimuth scaling will have an adverse effect on the subsequent feature extraction and target recognition. Since TAS does not consider the deviation of ERC caused by motion compensation, there are serious errors in the estimated effective rotational velocity (ERV). Its inaccuracy leads to inaccurate range spatial-variant autofocus, thus, the imaging results of IMCM-PGA-TAS are still blurred as shown in the dashed boxes. By contrast, with consideration for the deviation of equivalent rotational center (ERC), IMCM-PGA-MJAAS can jointly realize the accurate estimation of equivalent rotational center position (ERCP) and effective rotational velocity (ERV), thereby simultaneously performing precise azimuth scaling and range spatial-variant autofocus. Compared with Figure 3b, Figure 5 reveals that the proposed algorithm can obtain ISAR images with good focusing and accurate scaling at different SNRs. In this experiment, image entropy (IE) is used to evaluate image quality. It can be seen from the IE marked in Figure 5 that the proposed algorithm always achieves lower IE in different situations, which further quantitatively illustrates the superiority of the proposed algorithm. Table 3 shows the estimated ERV of IMCM-PGA-TAS and IMCM-PGA-MJAAS at different SNRs. Compared with real values, the proposed algorithm obtains more accurate estimation results at different SNRs, while large errors occur in the estimation results of TAS because it neglects the deviation of ERC. Figure 6 gives the ISAR imaging results of three algorithms in NCM as shown in Figure 4c,d at three SNRs, and Table 4 shows the ERV estimated by IMCM-PGA-TAS and IMCM-PGA-MJAAS at different SNRs. It can be seen from the dashed boxes and IE as well as the estimated sizes marked in the figure that the two comparison algorithms still have the same problems as they do in CM, while the proposed algorithm can achieve ideal imaging results at different SNRs. Table 4 also demonstrates that MJAAS has better estimation accuracy than TAS. Therefore, IMCM-PGA-MJAAS outperforms IMCM-PGA and IMCM-PGA-TAS, achieving better experimental results in different modes of motion errors at different SNRs. These experimental results are testament to the effectiveness and robustness of the proposed algorithm.

**Figure 7:**
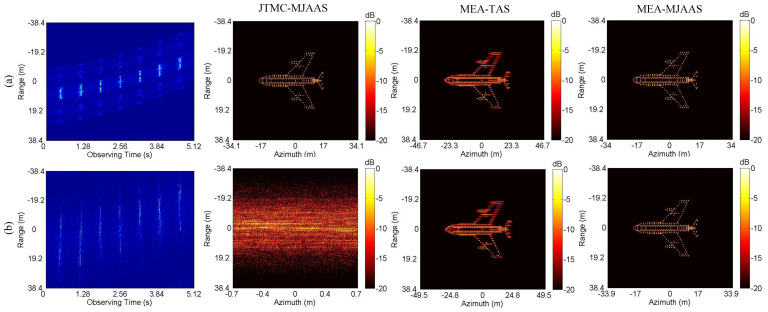
The imaging results of different algorithms corresponding to CM and NCM. (**a**) CM. (**b**) NCM.

The MJAAS proposed in this paper is applicable to not only the motion compensation algorithm (IMCM+PGA) adopted in this paper, but also other motion compensation algorithms, including the joint translational motion compensation algorithm (JTMC) in [20] and the range alignment and phase autofocus algorithms based on the minimum entropy (MEA) in [12,18]. Combined with TAS and MJAAS, three kinds of ISAR imaging frameworks are set up, namely JTMC-MJAAS, MEA-TAS and MEA-MJAAS. The three frameworks are adopted to produce ISAR images in CM and NCM when SNR = 20 dB, with the experimental results shown in Figure 7. It can be seen that since MEA-TAS neglects the deviation of ERC, the imaging results are defocused, and the scaling results are seriously distorted. JTMC-MJAAS can obtain ideal imaging results in CM, but not in NCM, because JTMC is not suitable for NCM. MEA-MJAAS can achieve satisfactory experimental results in both CM and NCM. With the same hardware utilized in the experiment, it takes 355 s and 11 s for MEA-MJAAS and IMCM-PGA-MJAAS to produce an image, respectively. This is because MEA-MJAAS needs to search and compensate the phase error of each echo, respectively; hence, there is a large amount of computation. In conclusion, the above experiments are testimony to the fitness of MJAAS proposed in this paper for different motion compensation algorithms. Moreover, the ISAR imaging framework adopted in this paper, IMCM-PGA-MJAAS, proves suitable for different modes of motion errors, thus, it has the advantages of strong practicality, high efficiency and high precision.

**Figure 8:**
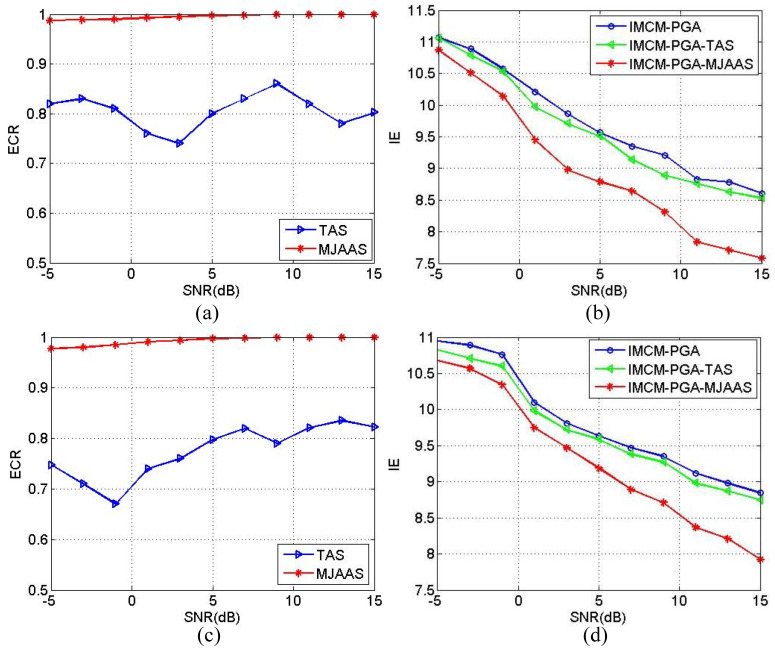
The relationship curves between ECR and SNR and the relationship curves between IE and SNR corresponding to CM and NCM. CM: (**a**) ECR; (**b**) IE. NCM: (**c**) ECR; (**d**) IE.

In order to quantitatively demonstrate the superiority of the proposed algorithm, the estimated correct rate (ECR) of ERV is defined as in Equation (18):

(18) $$ECR=\left(1-\frac{\left|\omega_{e}-{\hat{\omega }}_{e}\right|}{\left|\omega_{e}\right|}\right)\times 100\%$$

where $\omega_{e}$ denotes the theoretical value of ERV and ${\hat{\omega }}_{e}$ the estimated value of ERV. With other conditions unchanged, SNRs range from −5 dB to 15 dB in step of 2 dB. The Monte-Carlo experiment is implemented 20 times at each SNR, and the resulted average values of ECR and IE in 20 experiments are calculated and plotted in Figure 8. Figure 8a reveals the average values of ECR obtained by IMCM-PGA-TAS and IMCM-PGA-MJAAS in CM, and Figure 8b shows the average values of IE obtained by IMCM-PGA, IMCM-PGA-TAS and IMCM-PGA-MJAAS. Figure 8c,d are the results achieved in NCM. From Figure 8, the proposed algorithm can obtain higher ECRs and lower IE at different SNRs than the comparison algorithms no matter in CM or NCM. Therefore, the superiority of the proposed algorithm is quantitatively corroborated.

### 4.2. Real Data Experiments

**Figure 9:**
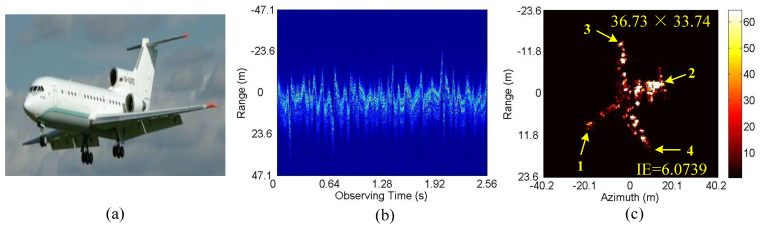
Real data of Yak-42 airplane. (**a**) Optical image. (**b**) HRRPs. (**c**) The ISAR image obtained by the proposed framework.

**Figure 10:**
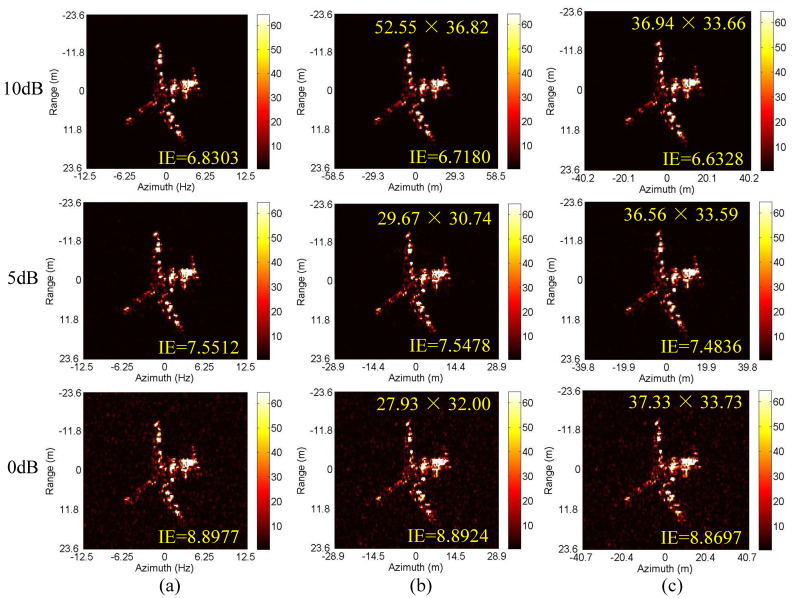
The imaging results of different algorithms at different SNRs. (**a**) IMCM-PGA. (**b**) IMCM-PGA-TAS. (**c**) IMCM-PGA-MJAAS.

Firstly, the comparison of performance between different algorithms is conducted based on the real data of the Yak-42 airplane. The main parameters of the radar system are as follows: carrier frequency is 5.52 GHz, bandwidth 400 MHz and PRF 50 Hz; the sampling point number of range and azimuth are 256 and 256, respectively. Moreover, the Yak-42 airplane is 36.38 m in length and 34.88 m in width. Figure 9a is the optical image of the Yak-42 airplane. Figure 9b,c are the envelope waveform of the echo signal and the 2-D image obtained by IMCM-PGA-MJAAS when SNR = 20 dB. According to Figure 9b, the mode of motion errors corresponding to the real data of the Yak-42 airplane is NCM. From Figure 9c, the ERV is estimated at 0.0085 rad/s. Based on this value, after azimuth scaling, the length and width of the target calculated by points 1, 2, 3 and 4 marked in the figure are 36.73 m and 33.74 m, respectively, which are close to the real values. Therefore, 0.0085 rad/s is considered as the reference value of ERV in the following experiments. As is the case with Figure 6, the imaging results of IMCM-PGA, IMCM-PGA-TAS and IMCM-PGA-MJAAS when SNR = 10 dB, 5 dB and 0 dB are shown in Figure 10. The ERVs estimated by IMCM-PGA-TAS and IMCM-PGA-MJAAS are presented in Table 5.

**Figure 11:**
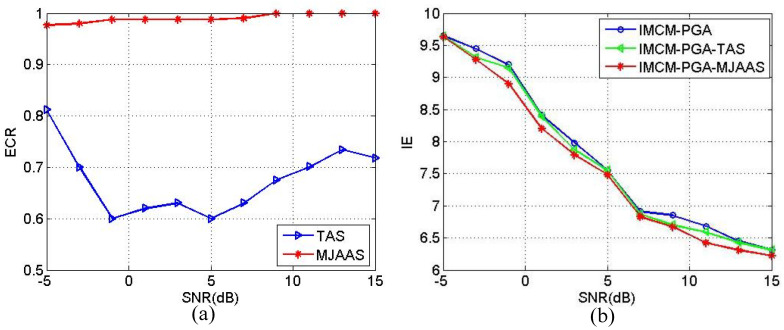
The relationship curve between ECR and SNR and the relationship curve between IE and SNR. (**a**) ECR. (**b**) IE.

In Figure 10, since IMCM-PGA does not compensate for the range spatial-variant phase errors of the target, the imaging results are defocused and the corresponding IE is high. Compared with Figure 9c, IMCM-PGA-TAS neglects the deviation of ERC, so there is a large error in the scaling results, which fail to reflect the real size of the target. IMCM-PGA-MJAAS obtains well-focused and accurately scaled ISAR images with lowest IE at different SNRs, laying a good foundation for subsequent feature extraction and target recognition. Moreover, Table 5 suggests that the ERV estimated by IMCM-PGA-MJAAS approximates to the real value, while that estimated by IMCM-PGA-TAS is far removed from the real value. In order to quantitatively demonstrate the superiority of the proposed algorithm, we adopt the method used in Figure 8c,d to calculate the average values of ECR and IE with the results shown in Figure 11. With no consideration for the deviation of ERC, serious errors arise in the ERV estimated by IMCM-PGA-TAS. By contrast, the ECR of the proposed algorithm remains more than 90% at different SNRs. Moreover, the proposed algorithm acquires lower IE than the two comparison algorithms at different SNRs. Therefore, these experiments quantitatively illustrate the superiority of the proposed algorithm.

**Figure 12:**
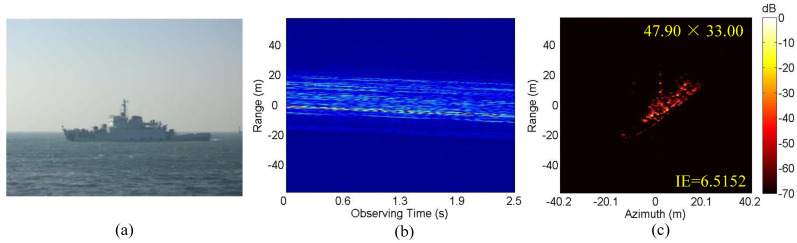
Real data of a ship. (**a**) Optical image. (**b**) HRRPs. (**c**) The ISAR image obtained by the proposed framework.

**Figure 13:**
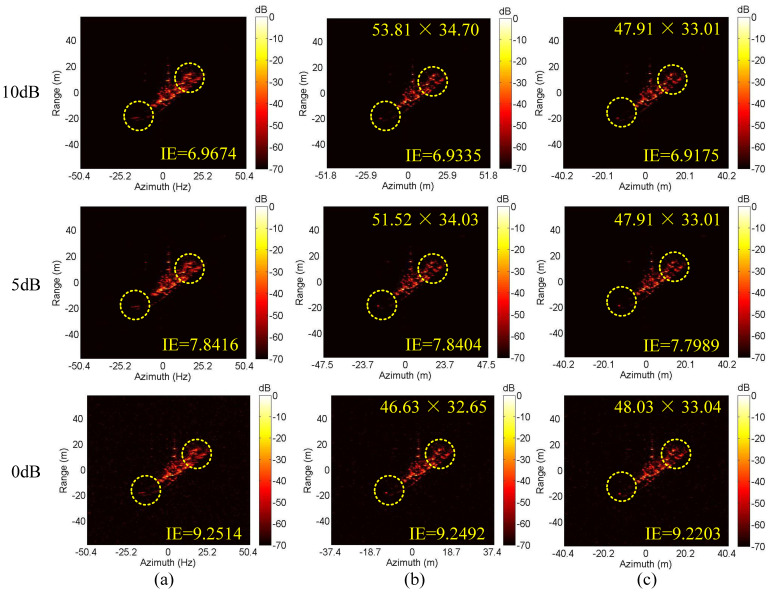
The imaging results of different algorithms at different SNRs. (**a**) IMCM-PGA. (**b**) IMCM-PGA-TAS. (**c**) IMCM-PGA-MJAAS.

**Figure 14:**
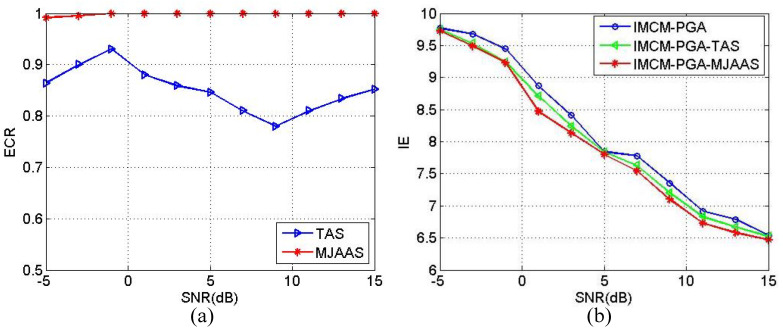
The relationship curve between ECR and SNR and the relationship curve between IE and SNR. (**a**) ECR. (**b**) IE.

Finally, we use a set of real data for a ship to further analyze the algorithm’s performance. The main parameters of the radar system are as follows: carrier frequency is 5.57 GHz, bandwidth 580 MHz and PRF 100.8 Hz; the sampling point number of range and azimuth are 448 and 256, respectively. Figure 12a is the optical image of the ship. Figure 12b,c are the envelope waveform of the echo signal and the 2-D image obtained by IMCM-PGA-MJAAS when SNR = 20 dB. According to Figure 12b, the mode of motion errors corresponding to the real data of the ship is CM. In Figure 12c, the estimated ERV of the ship is 0.0339 rad/s, which is used as the reference value. As is the case with Figure 5, the imaging results of IMCM-PGA, IMCM-PGA-TAS and IMCM-PGA-MJAAS when SNR = 10 dB, 5 dB and 0 dB are shown in Figure 13. The ERVs estimated by IMCM-PGA-TAS and IMCM-PGA-MJAAS are presented in Table 6. According to the dashed boxes and IE marked in Figure 13 and Table 6, the images obtained by IMCM-PGA are blurred, while the scaling results by IMCM-PGA-TAS contain serious errors. Under the same conditions, the proposed algorithm produces high-quality ISAR images, and the estimated values of ERV are basically consistent with the real value at different SNRs. These verify the effectiveness of the proposed algorithm. As can be seen in Figure 14, the proposed algorithm achieves more accurate ECRs and lower IE at different SNRs. Therefore, the above experiments quantitatively corroborate the superiority of the proposed algorithm.

## 5. Conclusions

This paper proposes a novel modified joint range spatial-variant autofocus and azimuth scaling algorithm (MJAAS). The motion compensation algorithm causes the deviation of ERC, which affects the estimation accuracy of ERV. This in turn results in the distortion of ISAR image azimuth scaling. To address this problem, the MJAAS algorithm is proposed to realize the joint estimations of ERCP and ERV by solving a minimum image entropy optimization problem via the DFP algorithm. Based on the estimation results, high-precision azimuth scaling and range spatial-variant autofocus can be achieved simultaneously so that high-resolution ISAR images with good focusing and accurate scaling can be obtained. Combined with IMCM and PGA, a complete ISAR imaging framework (IMCM-PGA-MJAAS) is established in this paper. It is not restricted by the modes of motion errors of the target and has low computational complexity, thereby having the advantages of strong practicality, high efficiency and high precision. A large amount of simulated and real data experiments can verify the superiority of the proposed algorithm. More efforts will be devoted to improving the performance of the proposed algorithm in the case of strong maneuvering targets with complex motions. Moreover, according to reference [30], artificial intelligence (AI) is expected to be integrated into our future work to improve the performance of the proposed algorithm, so as to achieve the ISAR imaging and azimuth scaling of high precision, strong robustness and low computational complexity.

## Figures and Tables

**Table 1:** Pros/cons of the four algorithms. TTM: trajectory tracking method; SBM: slope-based method; ACREM: azimuth chirp rates estimation method; MJAAS: modified joint range spatial-variant autofocus and azimuth scaling algorithm.

	Accuracy	Robustness	Computational Complexity	Affected by ERCP	Application Range
TTM	relatively low	relatively strong	low	no	relatively wide
SBM	relatively high	relatively low	relatively low	no	relatively narrow
ACREM	high	relatively low	relatively high	yes	relatively wide
MJAAS	high	strong	relatively high	no	wide

**Table 2:** Main Simulated Parameters. ERV: effective rotational velocity.

Parameters	Values
Carrier frequency	5.52 GHz
Bandwidth	500 MHz
Pulse repetition frequency	100 Hz
Pulse duration	20 μs
Sampling frequency	12.8 MHz
ERV ($\omega_{e}$)	0.04 rad/s
Range bin number	256
Azimuth bin number	512

**Table 3:** The estimated results of $\omega_{e}$ corresponding to CM (rad/s).

	Real Value	10 dB	5 dB	0 dB
TAS	0.04	0.0462	0.0320	0.0318
MJAAS	0.04	0.04	0.0399	0.0397

**Table 4:** The estimated results of $\omega_{e}$ corresponding to NCM (rad/s).

	Real Value	10 dB	5 dB	0 dB
TAS	0.04	0.0325	0.0481	0.0524
MJAAS	0.04	0.04	0.0401	0.0395

**Table 5:** The estimated results of $\omega_{e}$ (rad/s).

	Real Value	10 dB	5 dB	0 dB
TAS	0.0085	0.0058	0.0119	0.0118
MJAAS	0.0085	0.0085	0.0086	0.0084

**Table 6:** The estimated results of $\omega_{e}$ (rad/s).

	Real Value	10 dB	5 dB	0 dB
TAS	0.0339	0.0263	0.0287	0.0364
MJAAS	0.0339	0.0339	0.0339	0.0337

## Appendix A

In Equation (16), the one-order partial derivative of $\hat{\beta }$ is derived as (A1):

(A1) $$\frac{\partial IE}{\partial \hat{\beta }}=-\frac{1}{E_{g}}\sum_{n=-N/2}^{N/2-1}\sum_{k=-M/2}^{M/2-1}\left[\left(1+\ln\mu \right)\cdot \frac{\partial \mu }{\partial \hat{\beta }}\right]$$

where $\mu ={\left|g\left(n;k;\hat{\Psi }\right)\right|}^{2}=g\left(n;k;\hat{\Psi }\right)\cdot g^{*}\left(n;k;\hat{\Psi }\right)$. Then, we have Equation (A2):

(A2) ∂μ∂β^=g∗n;k;Ψ^⋅∂gn;k;Ψ^∂β^+gn;k;Ψ^⋅∂g∗n;k;Ψ^∂β^=2Reg∗n;k;Ψ^⋅∂gn;k;Ψ^∂β^

According to Equation (13), we have Equation (A3):

(A3) $$\frac{\partial g\left(n;k;\hat{\Psi }\right)}{\partial \hat{\beta }}=\frac{1}{M}\sum_{m=-M/2}^{M/2-1}\left\{\begin{array}{l} \exp\left(-j\frac{2\pi }{M}mk\right)\cdot \tilde{S}\left(n;m\right)\\ \cdot \exp\left\{-j\frac{4\pi }{\lambda }\cdot \left[\left(l_{n}-\hat{\beta }\right)\cdot \hat{\Omega }\cdot {\left(m\cdot \Delta t_{m}\right)}^{2}\right]\right\}\cdot \left[j\frac{4\pi }{\lambda }\cdot \hat{\Omega }\cdot {\left(m\cdot \Delta t_{m}\right)}^{2}\right] \end{array}\right\}$$

as is the case with the derivation of $\hat{\beta }$, we obtain the partial derivative of $\hat{\Omega }$, whose expression is expressed as Equation (A4):

(A4) $$\frac{\partial g\left(n;k;\hat{\Psi }\right)}{\partial \hat{\Omega }}=\frac{1}{M}\sum_{m=-M/2}^{M/2-1}\left\{\begin{array}{l} \exp\left(-j\frac{2\pi }{M}mk\right)\cdot \tilde{S}\left(n;m\right)\\ \cdot \exp\left\{-j\frac{4\pi }{\lambda }\cdot \left[\left(l_{n}-\hat{\beta }\right)\cdot \hat{\Omega }\cdot {\left(m\cdot \Delta t_{m}\right)}^{2}\right]\right\}\cdot \left\{-j\frac{4\pi }{\lambda }\cdot \left[\left(l_{n}-\hat{\beta }\right)\cdot {\left(m\cdot \Delta t_{m}\right)}^{2}\right]\right\} \end{array}\right\}$$

## Appendix B

The updated formula of H is shown in Equation (A5):

(A5) $$H^{\left[u+1\right]}=\left\{\begin{array}{cc} H^{\left[u\right]} & if\left[{\left(h^{\left[u\right]}\right)}^{T}\cdot x^{\left[u\right]}\leq 0\right]\\ H^{\left[u\right]}-\frac{H^{\left[u\right]}\cdot x^{\left[u\right]}\cdot {\left(x^{\left[u\right]}\right)}^{T}\cdot H^{\left[u\right]}}{{\left(x^{\left[u\right]}\right)}^{T}\cdot H^{\left[u\right]}\cdot x^{\left[u\right]}}+\frac{h^{\left[u\right]}\cdot {\left(h^{\left[u\right]}\right)}^{T}}{{\left(h^{\left[u\right]}\right)}^{T}\cdot x^{\left[u\right]}} & if\left[{\left(h^{\left[u\right]}\right)}^{T}\cdot x^{\left[u\right]}>0\right] \end{array}\right.$$

where u is η the iteration number, 0≤u≤U−1, and U is the total iteration number; $x^{\left[u+1\right]}={\hat{\Psi }}^{\left[u+1\right]}-{\hat{\Psi }}^{\left[u\right]}$, $h^{\left[u+1\right]}=\nabla IE\left[{\hat{\Psi }}^{\left[u+1\right]}\right]-\nabla IE\left[{\hat{\Psi }}^{\left[u\right]}\right]$. In particular, the initial value of the approximate matrix is set as $H^{\left[0\right]}=I_{2\times 2}$, where $I_{2\times 2}$ is the unit matrix.
